# Changing atmospheric acidity as a modulator of nutrient deposition and ocean biogeochemistry

**DOI:** 10.1126/sciadv.abd8800

**Published:** 2021-07-07

**Authors:** Alex R. Baker, Maria Kanakidou, Athanasios Nenes, Stelios Myriokefalitakis, Peter L. Croot, Robert A. Duce, Yuan Gao, Cécile Guieu, Akinori Ito, Tim D. Jickells, Natalie M. Mahowald, Rob Middag, Morgane M. G. Perron, Manmohan M. Sarin, Rachel Shelley, David R. Turner

**Affiliations:** 1 Centre for Ocean and Atmospheric Sciences, School of Environmental Sciences, University of East Anglia, Norwich, UK.; 2 Environmental Chemical Processes Laboratory (ECPL), Department of Chemistry, University of Crete, Heraklion, Greece.; 3 Center of Studies of Air quality and Climate Change, Institute for Chemical Engineering Sciences, Foundation for Research and Technology Hellas, Patras, Greece.; 4 Excellence Chair, Institute of Environmental Physics, University of Bremen, Bremen, Germany.; 5 Laboratory of Atmospheric Processes and their Impacts (LAPI), École Polytechnique Fédérale de Lausanne (EPFL), Lausanne, Switzerland.; 6 Institute for Environmental Research and Sustainable Development (IERSD), National Observatory of Athens (NOA), GR-15236 Palea Penteli, Greece.; 7 iCRAG (Irish Centre for Research in Applied Geoscience), Earth and Ocean Sciences, School of Natural Sciences and Ryan Institute, National University of Ireland Galway, Galway, Ireland.; 8 Departments of Oceanography and Atmospheric Sciences, Texas A&M University, College Station, TX, USA.; 9 Department of Earth and Environmental Sciences, Rutgers University, Newark, USA.; 10 Sorbonne Université, CNRS, Laboratoire d’Océanographie de Villefranche (LOV), Villefranche sur Mer, France.; 11 Yokohama Institute for Earth Sciences, JAMSTEC, Yokohama, Kanagawa, Japan.; 12 Department of Earth and Atmospheric Sciences, Cornell University, Ithaca NY, USA.; 13 Department of Ocean Systems (OCS), Royal Netherlands Institute for Sea Research, P.O. Box 59, 1790 AB Den Burg, Texel, Netherlands.; 14 Institute for Marine and Antarctic Studies, University of Tasmania, Hobart, Tasmania, Australia.; 15 Geosciences Division, Physical Research Laboratory, Ahmedabad, India.; 16 Department of Earth, Ocean and Atmospheric Science, Florida State University, Tallahassee, USA.; 17 Department of Marine Sciences, University of Gothenburg, Gothenburg, Sweden.

## Abstract

Anthropogenic emissions to the atmosphere have increased the flux of nutrients, especially nitrogen, to the ocean, but they have also altered the acidity of aerosol, cloud water, and precipitation over much of the marine atmosphere. For nitrogen, acidity-driven changes in chemical speciation result in altered partitioning between the gas and particulate phases that subsequently affect long-range transport. Other important nutrients, notably iron and phosphorus, are affected, because their soluble fractions increase upon exposure to acidic environments during atmospheric transport. These changes affect the magnitude, distribution, and deposition mode of individual nutrients supplied to the ocean, the extent to which nutrient deposition interacts with the sea surface microlayer during its passage into bulk seawater, and the relative abundances of soluble nutrients in atmospheric deposition. Atmospheric acidity change therefore affects ecosystem composition, in addition to overall marine productivity, and these effects will continue to evolve with changing anthropogenic emissions in the future.

## Teaser

Changing atmospheric acidity alters the delivery of nutrients to the ocean and affects marine productivity and ecology.

## INTRODUCTION

Atmospheric deposition supplies nutrients such as nitrogen (N), phosphorus (P), and iron (Fe) and other bioactive trace elements to the ocean in sufficient quantities to affect ocean productivity (*1*–*4*). Emissions of N species to the atmosphere are now dominated by anthropogenic combustion processes [nitrogen oxides (NOx)] and agricultural emissions [ammonia (NH_3_) and NOx]. Predominantly, natural mineral dust emissions are a major source of aerosols that contain a number of bioactive trace elements [e.g., P, Fe, copper (Cu), cobalt (Co), nickel (Ni), cadmium (Cd), and zinc (Zn)]. Industrial and biomass combustion sources also introduce Fe and other trace metals (e.g., Cu, Zn, Cd, and Ni) into the atmosphere [e.g., (*5*–*7*)], with ship emissions projected to become a major source of Fe to the North Atlantic and North Pacific oceans during the next century (*8*). Biomass burning has been suggested as a substantial source of pollutants to the global troposphere and specifically of nutrients to the remote ocean (*9*–*13*). Removal of these substances from the atmosphere occurs through dry and wet deposition. The balance between these removal processes is determined by the physical and chemical form (particularly whether in soluble or insoluble forms or as aerosol particles or gases) of the individual chemical species and by meteorology. These factors determine the atmospheric lifetime and geographic distribution of deposition.

Anthropogenic emissions of the acidic species NOx and sulfur dioxide (SO_2_) from fossil fuel combustion and of basic NH_3_ emissions from agriculture increased significantly after the Industrial Revolution. The effects of these emissions include the formation of acid rain (*14*) and a two- to threefold increase in the deposition of N to the oceans. This additional inorganic N supply is readily available to the marine microbial community, with resulting impacts on marine productivity (*2*, *15*). For many of the other elements considered here, some fraction of deposition is in the form of insoluble particulate phases that are not generally bioavailable. Although the chemical species that are bioavailable are often not clear, the term “labile” is used here to refer to the fraction of a given nutrient’s input that is potentially available to the microbial community upon deposition to the ocean. Observations suggest that the labile fractions of elements such as Fe and P increase during atmospheric transport, at least in part, due to exposure to acidic (i.e., low pH) conditions in aerosol and cloud waters (*16*–*18*). Similarly, the labile (soluble) fractions of a wide range of other bioactive trace metals such as Cu and Pb are also enhanced at low pH in rainwater (*19*).

**Fig. 1:**
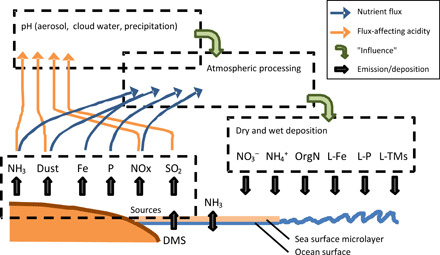
Overview of the atmospheric acidity, nutrient, and trace element cycles. Emissions of NH_3_, NOx, SO_2_, and dust influence atmospheric acidity (orange arrows). Dust, anthropogenic trace element emissions (abbreviated as Fe), and anthropogenic and biological sources of P, NH_3_, and NO_x_ contribute to the atmospheric nutrient/trace element burden (blue arrows). The majority of sources are terrestrial, although ship-based emissions of Fe and NOx are important and marine emissions of dimethyl sulfide (DMS) are a substantial source of SO_2_, particularly in the Southern Hemisphere. Acidity-driven atmospheric processing alters the labile nutrient flux to the ocean, either by affecting the gas-aerosol partitioning or by altering the labile fractions of Fe (L-Fe), P (L-P), and trace metals (L-TM). Organic nitrogen compounds (OrgN) are also generated during atmospheric processing but are not discussed here.

Anthropogenic emissions therefore have direct (N, P, and trace metals) and indirect (through their influence on atmospheric pH) impacts on the supply of labile nutrients to the ocean (Fig. 1). NOx and SO_2_ emissions are now decreasing or stabilizing, leading to regional-scale increases in rainfall pH (*20*), while ammonia emissions continue to increase (*21*, *22*). The overall effect of future emissions changes is projected to be a decrease in the acidity of aerosols, cloud water, and rainfall, as discussed later.

The atmospheric supply of available nutrients to the ocean has therefore changed in magnitude and distribution as a consequence of global industrialization and will continue to evolve over time with economic development and changes in regulatory emissions frameworks (*21*, *23*). These changes will occur in parallel with expected future changes in climate, for instance, changing humidity, rainfall amounts, and distribution (*24*), which will also affect the atmospheric nutrient delivery to the oceans through altered atmospheric lifetimes and changes in deposition mode as well as dust generation and biomass burning emissions (*25*–*27*). This work examines the combined impacts of atmospheric pH changes, brought about by evolving anthropogenic emissions, on labile nutrient deposition to the oceans and their expected effects on marine biogeochemistry.

## CONTROLS ON ATMOSPHERIC ACIDITY AND DRIVERS OF CHANGE

The IUPAC definition of pH for an aqueous solution is the negative logarithm of the hydronium ion (H_3_O^+^) activity on a molality basis. Aerosol thermodynamic models express aerosol particle pH in terms of per volume concentrations of hydronium and liquid water content (*28*)

$$\text{pH}=-{\text{log}}_{10}\gamma_{H^{+}}H_{\mathrm{aq}}^{+}=-{\text{log}}_{10}\frac{1000\gamma_{H^{+}}H_{\mathrm{air}}^{+}}{W}$$

where γ_*H*^+^_ is the hydronium ion activity coefficient, $H_{\text{aq}}^{+}$ (mol liter^−1^) is the hydronium ion concentration in the aqueous phase considered (aerosol or cloud water), $H_{\text{air}}^{+}$ (μg m^−3^) is the hydronium ion concentration per volume of air, and *W* (μg m^−3^) is the particle water concentration.

The two main drivers of pH are *W* and $H_{\text{aq}}^{+}$. *W* in aerosols is governed by relative humidity and hygroscopic properties of the aerosol conferred by the amount of soluble species present. For clouds, *W* is controlled by meteorology. $H_{\text{aq}}^{+}$ is controlled by the relative amounts and nature of dissolved ions (*28*). Sulfuric acid is the primary strong acid present in the atmosphere and is largely neutralized by gas-phase ammonia that partitions into the aqueous phase in the form of the ammonium ion (NH_4_^+^). Regionally, other nonvolatile cations (NVCs) found in sea salt and dust (e.g., K^+^, Na^+^, Ca^2+^, and Mg^2+^) also neutralize sulfuric acid–forming sulfate salts. The NVC content of dust is dependent on mineralogy (*29*). However, measurements have shown that submicron sea salt aerosol becomes acidic very fast once in the atmosphere by evaporation of water, capturing acidic gases and/or dissociation of organic compounds that can be assisted by sea salt cations (*30*). Gas-phase HNO_3_, HCl, and organic acids also partition to the aerosol/cloud phase and contribute to the acidity of the atmosphere (*28*), while amines may reduce acidity but are generally present in much lower amounts than NH_3_.

Fundamental differences exist between aerosol and cloud systems that lead to large differences in their respective acidity levels, and the associated response of cloud and aerosol pH to emissions trends. Cloud and rainwater constitute highly dilute solutions and exhibit pH levels that are many (up to four) units higher than in aerosol (*14*, *28*). In contrast, aerosol water is in chemical equilibrium with water vapor, its amount being determined by the ambient relative humidity (*14*). Thus, aerosol acidity is not controlled by the total amount of acidic and alkaline atmospheric loading but generally by the relative amounts of these species that exist in the aerosol phase and by temperature and relative humidity (*28*). Wintertime tends to yield aerosol with higher pH than summertime conditions due to the higher content of aerosol water driven both by relative humidity and aerosol chemical composition. High levels of NH_3_ associated with agriculture also elevate pH in Southeast Asia and North Europe [e.g., (*31*, *32*)], but aerosol remains mildly acidic over continental areas (see fig. S1).

The volatility of aerosol species is also important, because semivolatile species, such as NH_3_/NH_4_^+^, can largely reside in the gas phase even if the aerosol is strongly acidic (*33*). The presence of NVCs on larger particles elevates their pH, which increases the equilibrium vapor pressure of NH_4_^+^ and drives it to the gas phase in the form of NH_3_ [e.g., (*34*)]. The same increase in aerosol pH of large particles also promotes the partitioning of nitrates to the aerosol phase (*32*), which further elevates the aerosol pH because of the co-condensation of water. In contrast, the much lower pH of smaller aerosol particles is often driven by the thermodynamics of the ammonium-nitrate-sulfate system (*35*). These distributions cause the aerosol pH to vary considerably (generally increasing) with size, especially in the 1- to 2.5-μm particle diameter range (*28*, *30*, *36*, *37*). Therefore, acid processing of nutrients (e.g., Fe and P) is more effective on small particles that are more acidic and have a larger surface area–to–volume ratio than the coarse particles (*17*, *36*, *38*). Residence time and transport of particles increases as particle size decreases, implying that the greater load of labile Fe and P generated in a more acidic atmosphere is also transported over longer distances before deposition. The presence of organic compounds and their interactions with inorganic ions and aerosol water can also affect aerosol acidity [although evidence to date suggests that the effect on pH is only secondary (*34*, *39*)], while the pH, in turn, affects the partitioning of semivolatile organics and their dissociation into the aerosol phase (*28*).

Regional reductions in primary pollutant emissions have led to reduced acidity of cloud water and precipitation (*20*, *40*). Fine mode aerosol remains strongly acidic because of the volatilization of NH_3_ as aerosol sulfate concentrations decrease (*34*, *35*). This implies that strong fine mode aerosol acidity will persist into the future in many regions of the world (see fig. S1) (*41*). This stark contrast between the response of cloud and aerosol pH to emissions controls [e.g., (*42*)] carries implications for nutrient deposition that have not been fully appreciated to date and are further analyzed here.

## IMPACTS ON ATMOSPHERIC LABILE NUTRIENT CHEMISTRY

Atmospheric pH and the distribution of acidic and alkaline species have a number of influences on the transport of labile nutrients to the ocean, summarized in Fig. 1. Because the uptake of NH_3_ into the aerosol phase is driven primarily by the amount of available aerosol sulfate, decreases in sulfate lead to an increase in the proportion of the total NHx (= NH_3_ + NH_4_^+^) present as gaseous NH_3_. If the acidity is sufficiently low and the liquid water content is sufficiently high, then the partitioning of HNO_3_ to aerosol phase NO_3_^−^ is promoted—during which nitrate then becomes a controlling factor for the NHx partitioning as well (*43*). The atmospheric lifetimes of NH_3_ and NH_4_^+^ are significantly different, as is also the case for NO_3_^−^ and HNO_3_ (*14*, *22*, *44*); hence, aerosol acidity and liquid water changes (*43*) affect the long-range transport of NHx and total NO3 (= NO_3_^−^ + HNO_3_), as discussed below.

In some cases (notably Fe), the labile fraction of dust-associated elements in aerosols is extremely low near their sources (*45*, *46*) but increases due to the dissolution of inorganic mineral phases during long-range transport (*18*, *47*). The passage of dust particles through clouds appears to be important for this process, as it promotes the internal mixing of dust with acidic fine aerosols (*16*) and complexation of Fe with organic ligands (*48*, *49*) [mainly dicarboxylic acids that are produced during multiphase chemistry and contribute to atmospheric acidity (*50*)]. Labile Fe can also be produced in clouds by interaction with sulfur at aerosol surfaces (*51*) and as Fe-containing nanoparticles after surface dissolution (*52*). Lower pH conditions are required to solubilize Fe than to solubilize P (*17*, *48*, *53*). In addition, the susceptibility of nutrients to acid processing depends on their (geo)chemical forms. For instance, solubilization rates of ferrihydrite amorphous solids, nanosized iron oxides, and aluminosilicate crystalline forms show high, medium, and low dependence on aerosol acidity, respectively (*54*). Similarly, hydroxyapatite is more soluble than fluorapatite (*17*, *55*).

Total trace element amounts from combustion sources are often small in relation to those from mineral dust. However, combustion aerosols are dominated by fine mode particles that usually have high fractional solubility and are coemitted with acid precursor species [e.g., (*5*, *7*)]. Internal mixing of acidic fine mode sulfate with Fe-rich insoluble particles contained in combustion aerosols appears to enhance the dissolution of aerosol Fe and Cu (*36*). Results from chemical transport modeling indicate that the combination of very acidic conditions and high organic ligand concentrations contribute to sunstantial formation of labile Fe during atmospheric processing of combustion aerosols (*56*). Emissions of aerosol trace metals from combustion sources have also increased since preindustrial times and will change further into the future.

Strong complexation of Cu (*57*) and Fe with organic compounds such as siderophores (*58*) has been measured in rainwater. Relationships between Fe solubility and water-soluble organic carbon with Fe-binding functionalities (e.g., ─COOH and ─NH_2_) suggest a potential role of organic compounds in aerosols for sustaining high Fe solubility in solution (*59*, *60*). This role is less important under higher acidity conditions (*48*). Decreasing emissions of acid precursor gases are therefore expected to decrease the uptake of acidic species on mineral dust and combustion aerosol particles in the atmosphere and thus to lower the labile fraction of trace elements associated with these aerosols [e.g., Fe and P (*48*, *53*, *61*, *62*)].

Substantial uncertainties remain in our understanding of the processes that solubilize trace elements in the atmosphere and in atmospheric nutrient deposition in general. For instance, there is a wide range in Fe solubility in aerosols and rainwater over the Southern Ocean and Antarctica (*63*–*65*). Current models are not able to reproduce these observations, which suggest that they underestimate the labile Fe concentrations by up to a factor of 15 (*10*, *11*). The relative importance of the mechanisms capable of increasing the labile fraction of trace elements during transport (*66*) and the magnitude of labile trace element stabilization by organic complexation remain to be confirmed.

## IMPACTS OF ACIDITY CHANGES ON NUTRIENT DEPOSITION TO THE OCEAN

**Fig. 2:**
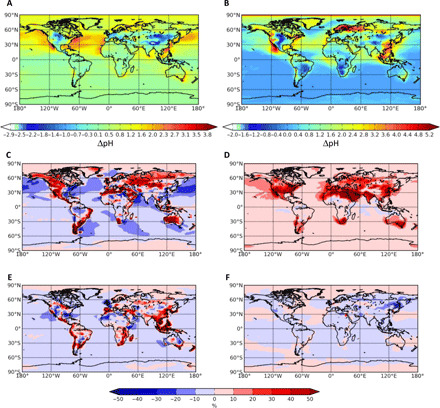
Impact of anthropogenic and biomass burning emissions changes (1850 to 2010) on aerosol pH and nutrient wet deposition fractions. Change in the annual mean near-surface (**A**) fine aerosol pH and (**B**) coarse aerosol pH and the change in the fractions of (**C**) wet NH_4_^+^ to the total NHx, (**D**) wet NO_3_^−^ to the total NO3, (**E**) wet L-Fe to the total L-Fe, and (**F**) wet L-P to the total L-P between 1850 and 2010. (C to F) The difference between 1850 and 2010, expressed as a percentage of the 2010 condition. Negative values denote higher values in 2010 than in 1850. Reference figures (aerosol pH and wet to the total deposition ratios) for 2010 and maps of the difference between 2100 and 2010 are provided in figs. S1, S4, and S5.

Changes in anthropogenic emissions since the Industrial Revolution have led to increasing acidity in fine and coarse aerosols, and these emissions continue to evolve in response to economic development and air pollution control measures. In the following, we use the Transport Model version 4 - Environmental Chemical Processes Laboratory (TM4-ECPL) global model of atmospheric deposition of nutrients (*67*) that has been evaluated against observations for the simulations of acidity (*28*) and the deposition fluxes of N (*53*, *68*), P (*53*), and Fe (*10*, *48*). Since the Industrial Revolution, aerosol acidity has increased by more than 1.5 pH units over broad regions of the midlatitude Northern Hemisphere ocean, with much larger pH decreases over the margins of the North Atlantic and North Pacific (Fig. 2, A and B, and fig. S1). These acidity changes have had a direct impact on the phase partitioning and deposition of N species (*22*, *33*, *43*). Higher acidity increases the proportion of reduced N in the form of the longer-lived, predominantly fine, aerosol NH_4_^+^ and decreases the proportion of gaseous NH_3_ (fig. S2C). Increased acidity reduces the partitioning of NO_3_^−^ to the aerosol phase (fig. S2D), reducing the lifetime of the total NO3 over most of the global ocean because of rapid removal of HNO_3_. These shifts in partitioning induce some notable changes in the overall NO3 deposition rate, particularly over the midlatitude Northern Hemisphere oceans, where increased acidity enhances its deposition rate as HNO_3_ and thus decreases lifetime (see fig. S3). The resulting longer lifetime for the total NHx and shorter lifetime for the total NO3 therefore affect their long-range transport, with NHx being transported over longer distances under acidic conditions, while NO3 is deposited closer to its terrestrial sources (*43*).

Changes in partitioning also affect the deposition mode for N, as illustrated by the changes in the ratio of wet to total deposition flux for NHx and NO3 (Fig. 2, C and D, and fig. S4). Thus, the total magnitude of the N deposition varies in response to emissions changes, while the spatial distribution of this deposition and its deposition mode vary as a function of atmospheric acidity. According to the TM4-ECPL simulations (*67*), the fraction of the total NO3 global deposition that is deposited to the ocean (total NO3 deposited to the ocean/global total NO3 deposition) has been reduced by about 10% since 1850 and will increase by about 5% in the future, following the gas-to-particle partitioning changes induced by the corresponding acidity changes over extended regions as shown in fig. S3. The equivalent fraction of the total NHx deposition entering the oceans decreased by 32% from 1850 to the present and is projected to decrease by a further 9% by 2100 (*67*), but these changes are predominantly due to the changes in NHx emissions rather than the changes in acidity, because, in almost all cases, NHx deposits with near gas-phase velocity.

The influence of acidity change on dust solubility affects the delivery of labile nutrients to the ocean. At present, 35% of the global total labile Fe flux is due to solubilization in the atmosphere, and the equivalent value for P is 32%. These values were 30 and 28% in the past (1850) and are expected to decrease to 20 and 26% by 2100, respectively (*48*, *53*). The global dissolution fluxes of the dust-associated labile Fe and P under 2005 anthropogenic and biomass burning emissions were estimated to be ~60% higher for Fe (*48*) and ~40% higher for P (*53*) than under 1850 emissions, while the overall labile Fe and P fluxes into the ocean increased by 34 and 27%, respectively. Decreases of similar magnitude in these fluxes (about 55 and 30% globally for the dissolution fluxes and 14 and 13% for the labile fluxes over the ocean of Fe and P, respectively) were projected to be reached by 2100, following the trend in pollutant emissions of the representative concentration pathways (RCP6.0).

However, the magnitudes of these changes are subject to substantial uncertainty, mostly related to the poor description in models of the aerosol mixing state (*50*), the adopted mineralogy, the mineral content for the respective nutrient and its form (*29*), and the emissions used (*67*). Uncertainties in deposition fluxes are also associated with the chemical scheme and the numeric solver used in the model (*69*), the thermodynamic modeling, and the pH calculations (*28*), as well as cloud and precipitation fields derived from meteorological reanalysis data (*10*). Changes in meteorology over time were not included in the simulations used in this study.

The different dependence of Fe and P solubilization on atmospheric acidity also alters the proportions of labile Fe and P in wet deposition (Fig. 2, E and F, and fig. S5) as a result of changes in hygroscopicity accompanying acidification of particle surfaces (*10*). The molar ratio of soluble Fe to soluble P in deposited dust aerosol [(Fe:P)_dust_] is also affected by changes in solubilization, with increases since 1850 in (Fe:P)_dust_ over the low latitude oceans where dust is mixed with biomass burning and anthropogenic emissions of acidic compounds and ligands (*50*). The differing responses of individual nutrients (soluble N, P, and Fe) to acidity changes therefore alters the N:P and N:Fe ratios of atmospheric deposition over the time scales considered here.

## IMPLICATIONS OF CHANGING AEROSOL ACIDITY ON NUTRIENT SUPPLY TO THE OCEAN

Materials deposited to the ocean must pass through the sea surface microlayer (SML), a transition zone whose composition is very different to that of bulk seawater (*70*, *71*). Residence times in the SML are spatially variable and of the order of a few minutes to hours (*72*, *73*), during which time deposited material is exposed to large gradients in pH and inorganic and organic complexing agents. Rainfall disrupts the SML (*74*) so that the deposition mode influences the extent to which atmospheric nutrient fluxes are modified (e.g., through changes in lability as a result of complexation) by transition through this interface zone. Shifts in deposition mode in response to changing acidity therefore also have the potential to affect the supply of labile nutrients to the ocean.

In oxygenated surface seawater, most nutrient and trace element species are significantly undersaturated with respect to mineral phases. Thus, most inorganic N is readily released upon deposition (*75*), while phosphate, which may be incorporated in iron oxyhydroxides or other minerals, is released more slowly (*76*). Iron, however, is poorly soluble (*77*, *78*), and its overall solubility under equilibrium conditions in seawater is controlled by the presence of organic complexes (*79*). Recent work has indicated that weaker ligands can be important in solubilizing Fe on short time scales of importance for scavenging reactions (*80*), and similar processes may work for Zn (*81*) and other metals. Atmospheric aerosols can contain substantial concentrations of carboxylic acids, which help to solubilize Fe in the aerosol/rain and could also slow the precipitation of Fe in seawater upon deposition (*82*–*84*). The presence of metal complexation in rain or aerosols (*58*, *60*) may increase the flux of trace metals to the dissolved phase upon deposition.

The availability of a particular element will depend on a competition between the pH-dependent formation of hydroxides and carbonates, the organic complexation of free metals, and dissociation of organic complexes present in the deposition (*85*, *86*). The importance of organic complexation of many metals in modifying their chemical speciation, solubility, and bioavailability in seawater is well documented, e.g., for Co (*87*), Cu (*88*), Cd (*89*), Fe (*90*), and Zn (*91*). Our knowledge of this organic binding has been gained from Competitive Ligand Exchange–Cathodic Stripping Voltammetry titrations (*92*). With few exceptions, these titrations have been carried out at buffered pH values close to that of seawater. Hence, the pH dependence of the metal-organic binding is poorly understood at present [see (*93*)]. The balance of the acidity and complexation-driven influences on solubility in seawater is uncertain and probably spatially very variable. However, the current global trend of the decreasing atmospheric acidity is occurring independently alongside ocean acidification (*94*), and therefore a continuing decline in the overall atmosphere–ocean pH gradient is to be expected. Current understanding of the role of the SML in modulating nutrient and trace element availability from atmospheric deposition is insufficient to predict the consequences of this change in pH gradient for oceanic nutrient availability.

The long-term N:P ratio in the ocean is controlled by plankton biogeography (*95*). On shorter time scales, however, change in the N:P supply from atmospheric deposition may alter the phytoplankton community and lead to wider ecosystem shifts over time. Analogous experiments looking at the impacts of altering the N:P ratio of upwelled waters in mesocosm experiments have seen significant changes in the taxonomy and nutritional quality of phytoplankton in the Peruvian upwelling (*96*, *97*). The changing of N:P and of Fe-to–macronutrient supply ratios may affect the colimitation relationships. The Fe requirements of a phytoplankton community depend on the N source, as NO_3_^−^ uptake or N_2_ fixation require more Fe than NH_4_^+^ uptake [~60% more for NO_3_^−^ reduction and ~7 to 11 times more for N_2_ fixation (*98*)]. Interaction between P and Fe, the nutrients usually assumed to govern N_2_ fixation, plays a crucial role in primary production and community composition (*99*, *100*). Model studies predict that changes in nutrient supply ratios can have large consequences for primary and export production as well as the community composition (*101*). For example, coupling the global N, Fe, and P deposition fluxes presented here to an ocean biogeochemistry model has indicated a shift from diatom to nanophytoplankton production in the northeastern Pacific, and an increase in primary production of 2.6% in the global ocean and up to 20% in the Northern Hemisphere subtropical gyres since 1850 (*102*).

## CONCLUSIONS AND RESEARCH PRIORITIES FOR THE FUTURE

Changing industrial, agricultural, and biomass burning emissions have had significant impacts on the acidity of the atmosphere (*28*), and this, in turn, influences the magnitude, spatial distribution and mode of deposition, and elemental ratios of labile nutrient supply to the ocean (*67*). These changes likely alter the manner in which the SML influences atmospheric nutrient transport to the ocean (*72*, *73*) and the impacts of nutrient deposition on marine microbial ecology (*102*). Future changes in emissions will continue to alter atmospheric acidity and nutrient deposition in the coming decades. The analysis presented here indicates that the acidity and liquid water content of atmospheric aerosol are key state parameters that profoundly influence the patterns, fluxes, and impacts of atmospheric nutrient deposition to the oceans.

There are many uncertainties in the influences of changing atmospheric acidity on nutrient inputs to the ocean. Accurate atmospheric chemical transport models are required to assess the magnitude and impacts of these changes, because the large spatial and temporal scales involved cannot be studied by observational programs alone. However, the development and validation of these models relies on the availability of appropriate high-quality observations (*10*). Current deficiencies in this research area include high-frequency, long-term monitoring of atmospheric composition over representative regions of the remote ocean (essential for understanding the impacts of long-term changes) (*103*) and observations of wet deposition (the dominant input across large areas of the global ocean but whose amounts and composition are very poorly sampled). Direct observations of aerosol pH have only recently become available [e.g., (*104*)]. Until these observations can be applied routinely to ambient aerosol, thermodynamic analysis of chemical composition of gas and aerosol, which includes observations of NHx and total NO3, provides the most reliable inference of aerosol pH (*28*). Such data are required in different aerosol size fractions and in regions with contrasting atmospheric chemical regimes (pollutants, mineral dust, terrestrial, and marine). Better understanding of trace element solubility in aerosols is also required, and measurements of solubility across a range of particle sizes that are linked to observations of aerosol pH may provide new insights into the processes that influence solubility. Rapid progress can also be expected from the fast-growing field of model/observation fusion—where in situ and satellite observations of air pollutants can be used to increase the accuracy of calculated deposition fluxes of nutrients and their impact estimated through biogeochemical modeling.

Improvements in understanding the role of the SML in the air-to-sea transfer of nutrients and trace elements are required, as is the inclusion of these processes in biogeochemical models. Knowledge of the complex interactions that lead to nutrient colimitation in marine microbial communities is limited (*105*). Predictions of the consequences for community composition of long-term changes in nutrient concentrations and/or supply ratios, or the evolution of community composition in response to those changes, should therefore be assessed carefully. Ocean acidification will also induce feedbacks on the acidity of the atmosphere, because ocean acidification will affect the air-sea exchange of ammonia, and is also expected to influence the production of gases such as dimethyl sulfide and volatile amines that alter the balance of acidity in the atmosphere (*106*).

## SUPPLEMENTARY MATERIALS

Supplementary material for this article is available at http://advances.sciencemag.org/cgi/content/full/7/28/eabd8800/DC1
